# Generalist Pollen-Feeding Beetles during the Mid-Cretaceous

**DOI:** 10.1016/j.isci.2020.100913

**Published:** 2020-03-18

**Authors:** David Peris, Conrad C. Labandeira, Eduardo Barrón, Xavier Delclòs, Jes Rust, Bo Wang

**Affiliations:** 1 Institute of Geosciences, University of Bonn, 53115 Bonn, Germany; 2 Department of Paleobiology, National Museum of Natural History, Smithsonian Institution, Washington, DC 20013, USA; 3 Department of Entomology and Behavior, Ecology, Evolution and Systematics Program, University of Maryland, College Park, MD 20742, USA; 4 College of Life Sciences, Capital Normal University, 100048 Beijing, China; 5 Museo Geominero, Instituto Geológico y Minero de España, 28003 Madrid, Spain; 6 Departament de Dinàmica de la Terra i de l’Oceà and Institut de Recerca de la Biodiversitat (IRBio), Facultat de Ciències de la Terra, Universitat de Barcelona, 08028 Barcelona, Spain; 7 State Key Laboratory of Palaeobiology and Stratigraphy, Nanjing Institute of Geology and Palaeontology and Centre for Excellence in Life and Palaeoenvironment, Chinese Academy of Sciences, 210008 Nanjing, China

**Keywords:** Biological Sciences, Evolutionary Biology, Evolutionary Ecology, Paleobiology

## Abstract

The Cretaceous fossil record of amber provides a variety of evidence that is essential for greater understanding of early pollination strategies. Here, we describe four pieces of ca. 99-million-year-old (early Cenomanian) Myanmar amber from Kachin containing four closely related genera of short-winged flower beetles (Coleoptera: Kateretidae) associated with abundant pollen grains identified as three distinct palynomorphotypes of the gymnosperm *Cycadopites* and *Praenymphaeapollenites cenomaniensis* gen. and sp. nov., a form-taxon of pollen from a basal angiosperm lineage of water lilies (Nymphaeales: Nymphaeaceae). We demonstrate how a gymnosperm to angiosperm plant-host shift occurred during the mid-Cretaceous, from a generalist pollen-feeding family of beetles, which served as a driving mechanism for the subsequent success of flowering plants.

## Highlights

• A gymnosperm to angiosperm plant-host shift is denoted during the mid-Cretaceous
• Kateretidae beetles are among the earliest pollinators of angiosperms
• Mesozoic direct evidences of angiosperm pollination just started to arise
• *Praenymphaeapollenites* is defined as a new angiosperm pollen morphotype

## Graphical Abstract

**Figure:**
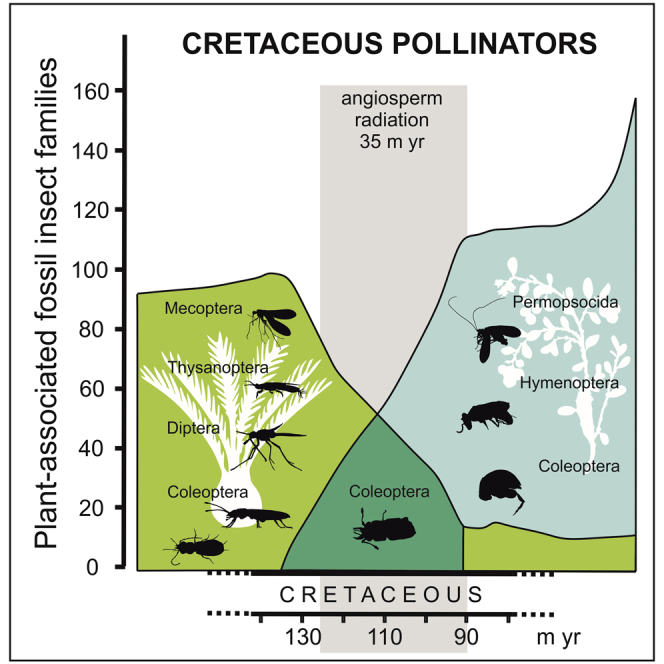

## Introduction

For much of land plant history, terrestrial vegetation consisted of free-sporing plants such as mosses, lycopods, ferns, horsetails, and gymnospermous seed plants whose mid-Mesozoic representatives included overwhelmingly conifers, cycads, ginkgoaleans, czekanowskialeans, corystosperms, caytonialeans, bennettitaleans, and gnetaleans (Labandeira et al., 2007, Labandeira, 2010, Friis et al., 2011). Angiosperms, currently the ecologically dominant and most diverse group (Feild and Arens, 2005), are a late-appearing seed-plant clade, commencing their diversification about 135 million years ago (mya) during the Valanginian, based on dispersed pollen data (Friis et al., 2011), although a macroscopic record of flowers and other reproductive structures began about 125 mya in the late Barremian (Friis et al., 2011). By the early Albian at about 112 mya, flowering plants came to dominate many habitats, representing a wide spectrum of lineages that were well differentiated by the end of the Early Cretaceous at 100 mya (Crane et al., 1995, Feild and Arens, 2005, Friis et al., 2006).

Mutualisms between insects and plants are among the most thoroughly studied of organismic interactions (Waser et al., 1996, Weiblen and Treiber, 2015). The origin of mutualisms and the conditions that foster their evolution is an enquiry that, for example, involves the origin of pollination in seed plants, especially angiosperms (Bernhardt and Thien, 1987, Kato and Inoue, 1994). This particular question principally has been addressed by two fundamental hypotheses that have been proposed regarding early angiosperm pollination biology during the Early Cretaceous (Taylor and Hu, 2010, Hu et al., 2012). The first hypothesis is that ancestral angiosperms were insect pollinated (entomophily) by a variety of generalized taxa (Bernhardt and Thien, 1987, Taylor and Hu, 2010, Endress and Igersheim, 2000, Thien et al., 2009). Accordingly, more derived, or specialized, modes of insect pollination appear during the Late Cretaceous or Paleogene (Crepet and Friis, 1987, Labandeira, 1998, Friis et al., 2006; but see Gandolfo et al., 2004). The second hypothesis states that the earliest angiosperm flowers were pollinated both by insects and abiotic mechanisms such as wind and possibly water (ambophily) (von Balthazar and Endress, 1999, Thien et al., 2009). These two hypotheses have been addressed in three fundamental ways: (1) various parsimony-based analyses of flower types and their pollen based on an incomplete, early angiosperm record (Taylor and Hu, 2010, Hu et al., 2012, Hu et al., 2008); (2) assessments of the relevant fossil insect record (Labandeira, 2010, Peris et al., 2017); and (3) contemporaneous pollination ecology studies applicable to the Early Cretaceous (Bernhardt and Thien, 1987, Endress and Igersheim, 2000, Thien et al., 2009, Endress, 2010).

Although wind pollination is the prevalent mode of pollination in extant gymnosperms, field observation and experimental studies have documented insect visitation in a number of cycad species and in all genera of Gnetales (Norstog, 1987, Schneider et al., 2002, APG, 2009). Endress (2010) noticed that nearly all bisexual flowers of basal angiosperms are protogynous, in that female reproductive organs come to maturity before the male organs. Consequently, protogyny was considered an ancestral trait in flowering plants (Sargent and Otto, 2004). Initially, protogyny was correlated with abiotic pollination (Endress, 2010); however, at present, strict wind pollination is uncommon in basal angiosperms and rather is a condition that often is shared with biotic pollination (Thien et al., 2009, Hu et al., 2008, Endress, 2010). A phylogenetic analysis of basal angiosperm pollinators shows that wind pollination is derived and has evolved numerous times during the mid-Cretaceous (Sargent and Otto, 2004, Hu et al., 2008, Hu et al., 2012). The thesis of ancestral insect pollination followed by derived modes of wind pollination is also supported by the earliest fossil record of angiosperm pollen, from the Late Valanginian through Aptian stages, before stickiness in pollen evolved (Sargent and Otto, 2004, Krassilov et al., 2005). Ambophily is understood as an unspecialized, ancestral condition that may have been an important pollination strategy for early angiosperms in newly invaded habitats during the Aptian, from which either specialized insect or wind pollination could have evolved (Hu et al., 2012). During the Albian, evolution of several pollination modes occurred (Crepet and Friis, 1987, Labandeira, 1998, Friis et al., 2006, Peris et al., 2017), and suites of flower traits evolved in response to conditions favoring greater efficiency of pollination. These traits included pollen clumping and floral nectaries, implying an increase in specialized zoophilous and wind pollination modes (Hu et al., 2008, Hu et al., 2012, Taylor and Hu, 2010).

In this report, we provide the first direct evidence that closely related species of short-winged flower beetles (Coleoptera: Kateretidae) fed on and transferred pollen from three different gymnosperm plant hosts and an early angiosperm plant host at the Early Cretaceous-Late Cretaceous boundary interval (99 mya). This breadth of pollinated seed plants by a lineage of closely related beetle taxa constituted an initial step in the transition from a gymnosperm-beetle to an angiosperm-beetle mutualism by a simple shift of a host plant from a gymnosperm to an angiosperm. The gymnosperm hosts (as pollen) were cycads, ginkgoaleans, and bennettitaleans, whereas the angiosperm host (also as pollen) was a water lily (Nymphaeales: Nymphaeaceae). Nymphaeales share a sister group or an adjacent paraphyletic relationship with Amborellaceae, the basalmost extant lineage of angiosperms. The closeness of these mutualisms provides a major advance for understanding the mode in which the transition from gymnosperm hosts to a basal angiosperm occurred in one particular insect pollinator family of beetles within the same local environment. We note that, to our knowledge, this is also the first evidence of a fossil association likely engaged in aggregative behavior.

## Results

**Figure 1:**
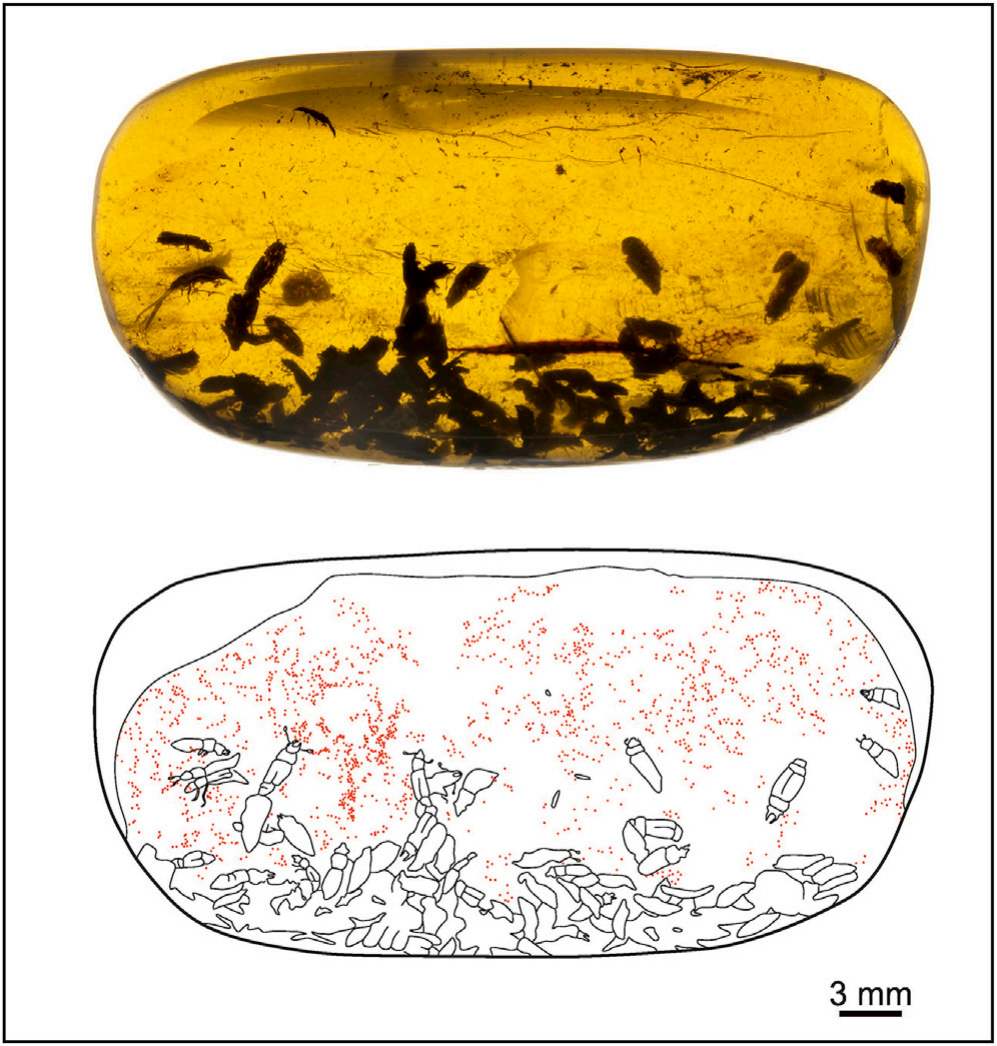
Light Microscope Photograph and Facsimile Camera Lucida Drawing of the Amber Sample NIGP171365 Each pollen grain is represented by a red dot placed in its observed position from the amber sample.

**Figure 2:**
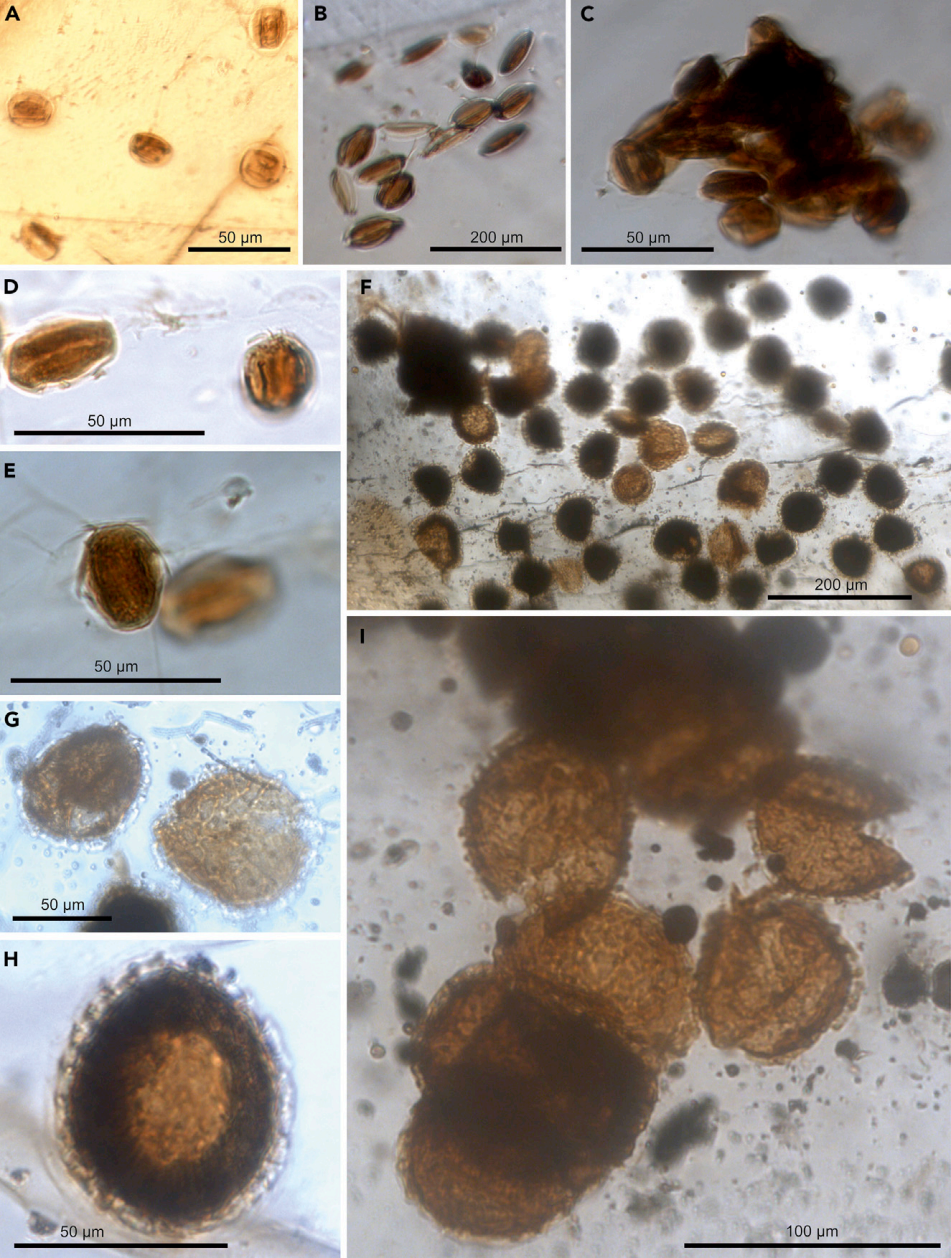
Pollen Grains Preserved within the Amber Samples (A) *Cycadopites* consisting of subprolate/rounded grains from sample MGB 87960, comparable with several palynospecies of Bennettitales. (B) Prolate grains of *Cycadopites* from Sample MGB 87961, affiliated with Cycadales and Ginkgoales. (C–E) Prolate grains of *Cycadopites* from sample NIGP171364, also affiliated with Cycadales and Ginkgoales, forming an intact clump in (C). (F–I) Rounded zonasulculate grains of sample NIGP171365, described herein as *Praenymphaeapollenites cenomaniensis* gen. and sp. nov., a newly defined angiosperm pollen, forming a clump in (I). See Figures S1 and S3.

Four pieces of Myanmar amber from Kachin (Figures 1 and S1–S3) were dated to the early Cenomanian (ca. 99 mya) of the Late Cretaceous (Shi et al., 2012) and later examined for their seed-plant and insect inclusions. Each piece contained a varied number of fossil beetles that were assigned to four different species of Kateretidae: *Electrumeretes birmanicus*, *Polliniretes penalveri*, *Cretaretes minimus*, and *Eoceniretes antiquus* (see Peris and Jelínek, 2019, Peris and Jelínek, 2020). We also observed abundant gymnosperm pollen grains of *Cycadopites* in three of the samples: MGB 87960, MGB 87961, and NIGP171364. The gymnosperm pollen grains are monosulcate, usually prolate to subprolate in shape, although many grains of sample MGB 87960 are rounded (see Systematic Paleontology). The prolate grains of the specimens in samples MGB 87961 and NIGP171364 could be affiliated with Cycadales and Ginkgoales (Figures 2B–2E), whereas the subprolate/rounded grains of the sample MGB 87960 are more comparable with several species of Bennettitales (Figure 2A). The occurrence of *Cycadopites* grains with three different morphologies associated with the beetles suggests the existence of multiple gymnosperm host plants.

The last sample (NIGP171365) that we examined, in contrast to the other pollen samples containing *Cycadopites* pollen, had pollen assigned to *Praenymphaeapollenites* gen. nov., a newly defined angiosperm pollen morphotype (Figures 2F–2I). This affiliation is notable as representing the only example of direct evidence of an aggregative insect-plant pollination association during the Cretaceous. All documented Mesozoic pollination mutualisms to date are between isolated insect specimens and plant hosts, a pattern present in several major deposits and representing a variety of pollination modes (Peris et al., 2017, Grimaldi et al., 2019).

**Figure 3:**
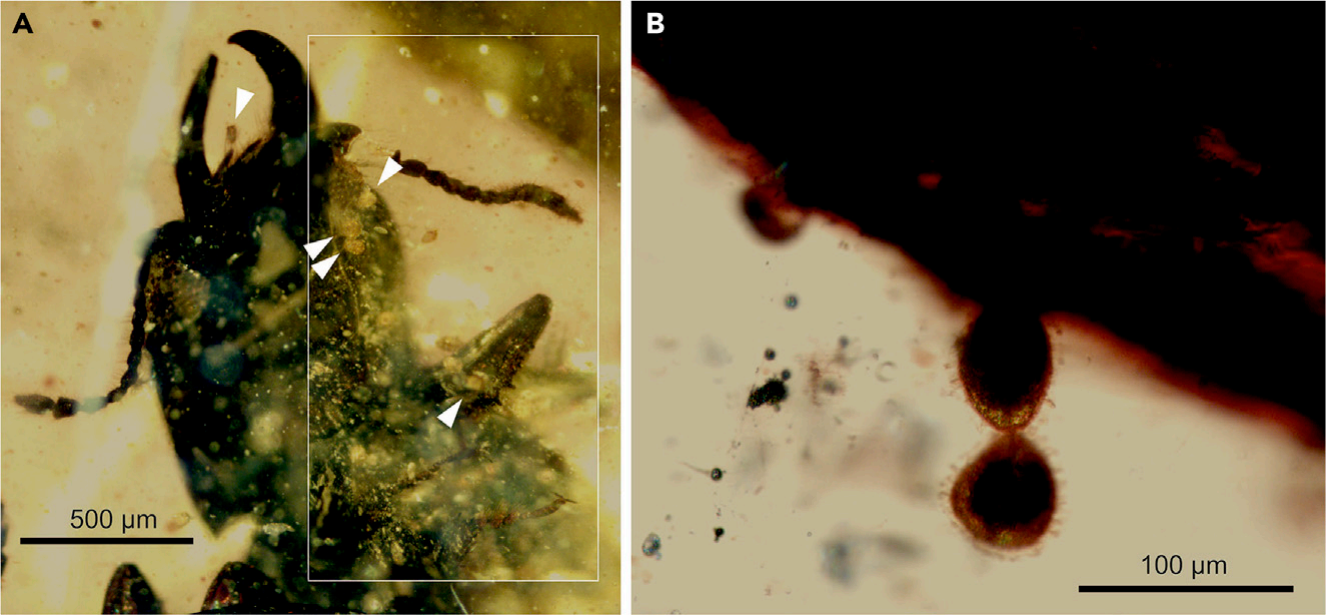
Pollen Grains Attached to the Body Surfaces of Kateretid Beetles (A) *Praenymphaeapollenites cenomaniensis* gen. and sp. nov. grains attached to the head and right proleg, indicated by white arrows, in a specimen from sample NIGP171365. The vertical rectangle delimits an area with better image resolution. (B) *P*. *cenomaniensis* sp. nov. grains attached to the elytron of a specimen from sample NIGP171365.

The number of beetles in each sample is highly variable. *Electrumeretes* *birmanicus* occurs isolated as one beetle in NIGP171364 together with an indeterminate beetle specimen as syninclusion (Peris and Jelínek, 2019; Figure S3); 41 beetles of *C*. *minimus* and 41 more specimens of *E*. *antiquus* occur in MGB 87960 and MGB 87961 (Peris and Jelínek, 2020; Figure S3), and somewhat more than 50 specimens of *P*. *penalveri* are present in NIGP171365 (Peris and Jelínek, 2019; Figure 1). The pollen grains in the samples are abundant and widely distributed within each sample, such that the pollen both surround and are in contact with the body of the beetle specimens (Figure 3). Importantly, some pollen grains are aggregated into distinct clumps (Figures 2C and 2I). Because (1) pollen consumption often has been the evolutionary precursor to pollination (Labandeira, 1998), (2) polylectic mutualism is found in fossil kateretids with a variety of gymnosperm hosts and one angiosperm host, and (3) the angiosperm pollen morphotype found belongs to a basalmost phylogenetic position, we postulate that Kateretidae was one of the first generalist pollinator lineages to access the earliest appearing angiosperms as hosts.

### Systematic Paleontology

Gymnospermae Lindley, 1830.

*Cycadopites* sp1 (MGB 87960) (Figure 2A)

*Description*: Pollen grains subprolate to rounded; 20.89 μm in length × 18.42 μm in width on average; range in length: 18.57–22.85 μm; range in width: 14.28–22.85 μm; monosulcate; sulcus elongate for the entire length of the grain; sulcus margin folded; exine; 1–2.5 μm thick; surface psilate.

*Cycadopites* sp2 (MGB 87961) (Figure 2B)

*Description*: Pollen grains prolate; 18.41 μm in length × 10.41 μm in width on average; range in length: 15.71–21.42 μm; range in width: 7.14–12.85 μm; monosulcate; sulcus elongate for the entire length of the grain, sometimes wider at their ends and constricted toward their equatorial area; sulcus margin folded; exine around 1.5 μm thick; surface psilate.

*Cycadopites* sp3 (NIGP171364) Figures 2C–2E

*Description*: Pollen grains prolate, sometimes subprolate; 26.27 μm in length × 14.84 μm in width on average; range in length: 21.42–32.14 μm; range in width: 10.71–17.85 μm; monosulcate; sulcus elongate for the entire length of the grain; sulcus outline oval, sometimes wider at their ends and constricted toward their equatorial area; sulcus margin folded; exine 1.5–2 μm thick; surface psilate. Pollen grains apparently are isolated or integrated in clusters of more than 15 specimens (Figure 2C).

*Remarks*: Modern Cycadales exhibit elongate, boat-shaped, longitudinally monosulcate and bilaterally symmetrical pollen grains (Dehgan and Dehgan, 1988), a characterization that almost is coincident with the diagnosis of fossil *Cycadopites* pollen (Fensome, 1983). At present, the pollen type *Cycadopites* is produced only by Cycadales and by the relict taxon *Ginkgo biloba*. However, fossil *Cycadopites* occurs in the pollinate organs of cycadaleans, bennettitaleans, ginkgoaleans, czekanowskialeans, peltaspermaleans, pentoxylaleans, and gnetaleans (Balme, 1995), representing the several, major, seed-plant lineages of the Mesozoic. Several of these lineages already were associated with insects in various ways throughout the Mesozoic (Labandeira et al., 2007).

Pollen grains of the samples MGB 87961 and NIGP171364 morphologically are similar to grains associated with *Gymnospollisthrips* (Thysanoptera) from late Albian amber of northern Spain (Peñalver et al., 2012) and with the pollen associated with a specimen of the scorpionfly taxon *Parapolycentropus paraburmiticus* in Cenomanian Myanmar amber (Lin et al., 2019). However, both records show pollen grains that are slightly smaller, 20 × 12.6 μm on average for the thrips and 12.15 × 7.17 μm on average for the scorpionfly, than the pollen described in this work. The *Cycadopites* pollen grains associated with the beetle *Cretoparacucujus cycadophilus*, from Cenomanian Myanmar amber (Cai et al., 2018), are similar in size to the pollen found adjacent to our kateretid specimen NIGP171364. The occurrence of a well-differentiated margo encircling the sulcus in pollen associated with *C*. *cycadophilus* (Figure 2) (Cai et al., 2018) evidently indicates the existence of different gymnosperm pollen producers in early Cenomanian ecosystems of northern Myanmar.

Angiospermae Linneaus, 1735, emend. de Candolle, 1824; Nymphaeales Dumortier 1829; Nymphaeaceae Salisbury, 1805.

*Praenymphaeapollenites* gen. nov. Barrón, Peris and Labandeira (NIGP171365) (Figures 2F–2I and 3)

*Type species*: *Praenymphaeapollenites cenomaniensis* gen. and sp. nov. Barrón, Peris and Labandeira.

*Etymology*: The generic name is derived from the prefix “*Prae*–*”* indicating a previous find, and the root “–*nymphaeapollenites,”* the most similar fossil pollen type, known only from the Cenozoic.

*Diagnosis*: Pollen grains zonasulculate, outline of the equator circular to subcircular, exine tectate, and strongly columellate; surface covered by evenly spaced thick verrucae as well as scarce short baculae sometimes ordered as an irregular reticulum-like pattern.

*Description*: Pollen grains zonasulculate, usually grains show broken sulculus being divided in two halves; operculum is lacking or cannot be observed; grains isolate or aggregate in clusters; outline of the equator circular to subcircular; longer equatorial axis 60.67 μm on average (range 42.85–85.71 μm); exine tectate and strongly columellate; endexine thin, 1–2 μm thick; ectexine columellate around 3–5 μm thick; columellae >1 μm width; thick verrucae usually appears in the surface at the top of each columellae; verrucae and in lesser extent baculae ornamentation; surface covered by spaced thick verrucae as well as scarce short bacula sometimes ordered in irregular reticulum-like pattern; verrucae 1–2 μm in basal width.

*Praenymphaeapollenites cenomaniensis* sp. nov. Barrón, Peris and Labandeira (Figures 2F–2I and 3)

*Etymology*: The specific epithet “*cenomaniensis”* refers to the Cenomanian age of the Kachin amber.

*Holotype*: NIGP171365. The sample is deposited in the Nanjing Institute of Geology and Palaeontology, Chinese Academy of Sciences, Nanjing, China.

*Type locality and age:* Tanai (= Danai), Hukawng Valley, Noije Bum Range, from Kachin State, northern Myanmar; Late Cretaceous (Early Cenomanian) in age (Shi et al., 2012).

*Diagnosis*: As for the genus, additional characteristics include the following: longer equatorial axis 60.67 μm on average (range 42.85–85.71 μm); endexine thin, 1–2 μm thick; ectexine columellate around 3–5 μm thick; columellae >1 μm width; verrucae 1–2 μm in basal width.

*Description*: As for the genus, measurements of the pollen are in Table S1.

*Remarks*: The pollen examined in NIGP171365 resembles grains of extant species of *Nymphaea* such as *Nymphaea alba* and *Nymphaea odorata*. This pollen also shows similarities in shape and aperture structure to the Neogene species described as *Nymphaeacidites* by Sah (1967) in Bore Hole Ru. 231 from Rusizi Valley in Burundi. However, the Cretaceous specimens that have been studied differ by their exine features that display thick, regular columellae and absence of reticulate ornamentation, blunt bacula, and spines. Nagy (1969) found spherical, zonasulculate, and echinate specimens in the Miocene of the Mecsek Mountains in Hungary that included the form genus *Nymphaeapollenites*. Although Thiele-Pfeiffer (1981) emended *Nymphaeapollenites*, by considering only pollen grains with psilate surfaces from zonasulcate specimens of the German Miocene, Nagy (1985) pointed out that *Nymphaeapollenites* is well differentiated from *Nymphaeacidites* by the lack of reticulate ornamentation. Cenozoic and recent grains are clearly distinguished from the examined Cretaceous ones by their finely columellate exines. Doyle (2005) considered that the exine structure in recent *Nuphar* and Nymphaeoideae, which is “intermediate” between the granular and columellar condition, was derived from a columellar pollen. The presence of a tectate-columellate exine with supratectal verrucate and baculate surface ornamentation in the examined specimens allows us to infer that *P*. *cenomaniensis* sp. nov. was a precursor taxon to extant Nymphaeaceae.

The oldest fossil record of nymphaealean plants is supported by Early Cretaceous leaves, flowers, and fruits (Friis et al., 2011). For pollen, the earliest record of dispersed pollen assigned to Nymphaeaceae corresponds to monosulcate, elliptical, and retipilate to reticulate pollen grains of late Maastrichtian age from the Maastrichtian-Paleocene Scollard Formation of Alberta, Canada (Srivastava, 1969, Muller, 1981). The material described here is the oldest nymphaealean pollen grain presently described. Although Nymphaeaceae is a basal family of angiosperms (APG, 2009; although see Discussion), no fossil angiosperm vegetative or reproductive material preserved as bioinclusions in Cenomanian Myanmar amber could be affiliated with this clade. At present, only angiosperm mesofossils or pollen of eumagnoliids, monocots, and eudicots are described from this amber (Poinar, 2004, Chambers et al., 2010, Poinar et al., 2016, Mao et al., 2018, Soltis et al., 2018).

## Discussion

### Insect Pollination during the Cretaceous

Occasionally, the fossil record reveals evidence of pollinivory, or pollen consumption, which consists of pollen present in insect guts, or more remotely, pollen found in coprolites in which the identities of the insect consumer and consumed pollen often are known. However, pollinivory is not coextensive with pollination, as some palynivores are not pollinators, and vice versa. Nevertheless, pollen consumption often has been the evolutionary precursor to biotic pollination (Labandeira, 1998). Alternatively, direct evidence can involve an insect in close or intimate association with pollen grains that evidently it is transporting (Wappler et al., 2015), whether or not pollen is consumed. Such pollen transport by an insect is facilitated by attachment of pollen to a receptive body part, particularly mouthparts, head, and legs, but occasionally other parts of their body, including specific pollen capture structures. It is impossible from fossil material to determine if the pollen grain of a plant associated with or otherwise consumed by an insect ever reached and penetrated the stigma of a conspecific individual that allowed for successful reproduction. In many instances, our examined amber material contained abundant pollen grains that were widely distributed within the amber. Given this record of scattered dispersal of pollen in amber specimens that also contained insects, it would be unjustified to define such insects commingled with pollen as pollinators. Widely strewn grains more likely would have been present in the resin simply by chance occurrence. Accordingly, such an insect would lack a functional relationship with nearby pollen grains because of their absence of external hair vestiture for entrapping pollen, an appropriate mouthpart morphology for processing pollen, or other distinguishing features typical of insect pollinators such as pollen baskets. Happenstance occurrences between an insect and scattered pollen in the same piece of amber occasionally occur in amber, particularly where a beetle taxon lacks evidence for consumption of the adjacent pollen grains or involvement in a pollination mutualism. Consequently, it would be inappropriate to ascribe a distinctly new pollination habit for the insect species and its surrounding host-plant species represented by pollen grains, without confirming with indirect or especially direct evidence. Two issues are relevant. First, one salient feature important in such amber material is whether the pollen grains are preserved in association with the insects and have contact with parts of the insect's body surfaces that suggest a function of transport, feeding, or other interaction. Second, additional confirmation would include, such as the examined specimens of this work, documentation that a pollination interaction is present among the descendant insect lineage in their interactions with their current host-plants. If these two criteria are satisfied, one can posit a reasonable hypothesis that such insects in the distant past were acting as pollinators.

Pollination relationships among diverse insect and gymnosperm groups since the Late Paleozoic have been suggested by substantial but indirect paleontological evidence (Ren et al., 2009, Labandeira, 2010, Khramov and Lukashevich, 2019). Indirect evidence from seed plants consists of structures such as tubes, funnels, channels, micropyles, and salpinx apertures in ovulate organs of gymnosperms designed to receive the mouthparts of long-proboscid insect pollinators (Ren et al., 2009, Labandeira, 2010), or the presence of structures, such as floral nectary discs, in angiosperms that attract mandibulate insect pollinators (Friis, 1985). Additional support is provided by entomophilous features of fossil pollen, such as size, shape, ornamentation, stickiness, quantity, and clumping ability (Taylor and Hu, 2010, Hu et al., 2012). For insects, the specialized modification of mouthparts into elongate siphonate proboscises commonly is used to access pollination drops in tubular ovulate organs of gymnosperms or the deep-throated flowers of angiosperms (Nilsson, 1998, Ren et al., 2009). The variety of long-proboscid brachycerous flies (Diptera), aneuretopsychine scorpionflies (Mecoptera) and kalligrammatid lacewings (Neuroptera) found in the pre-angiosperm fossil record (Ren et al., 2009, Labandeira, 2010, Peñalver et al., 2015, Labandeira et al., 2016, Lu et al., 2016, Peris et al., 2017, Liu et al., 2018, Lin et al., 2019) consequently provides indirect morphological evidence for pollinator activity. This evidence occurs individually either on the plant host or on the insect pollinator, but without the direct associational evidence of a plant structure adjacent to or in contact with an insect, such as pollen occurring in the pollen basket of a bee (Wappler et al., 2015). Evidence from fossil gut contents and coprolites of pollen-consuming insects (Labandeira, 1997, Labandeira, 1998, Labandeira et al., 2007) (1) can identify the plant source from palynology; (2) can identify the association, in that pollen has been consumed by an insect; but (3) almost always cannot identify the insect palynivore. Unless the identity of the insect that produced the coprolite can be made, a very rare circumstance, it constitutes indirect evidence.

**Figure 4:**
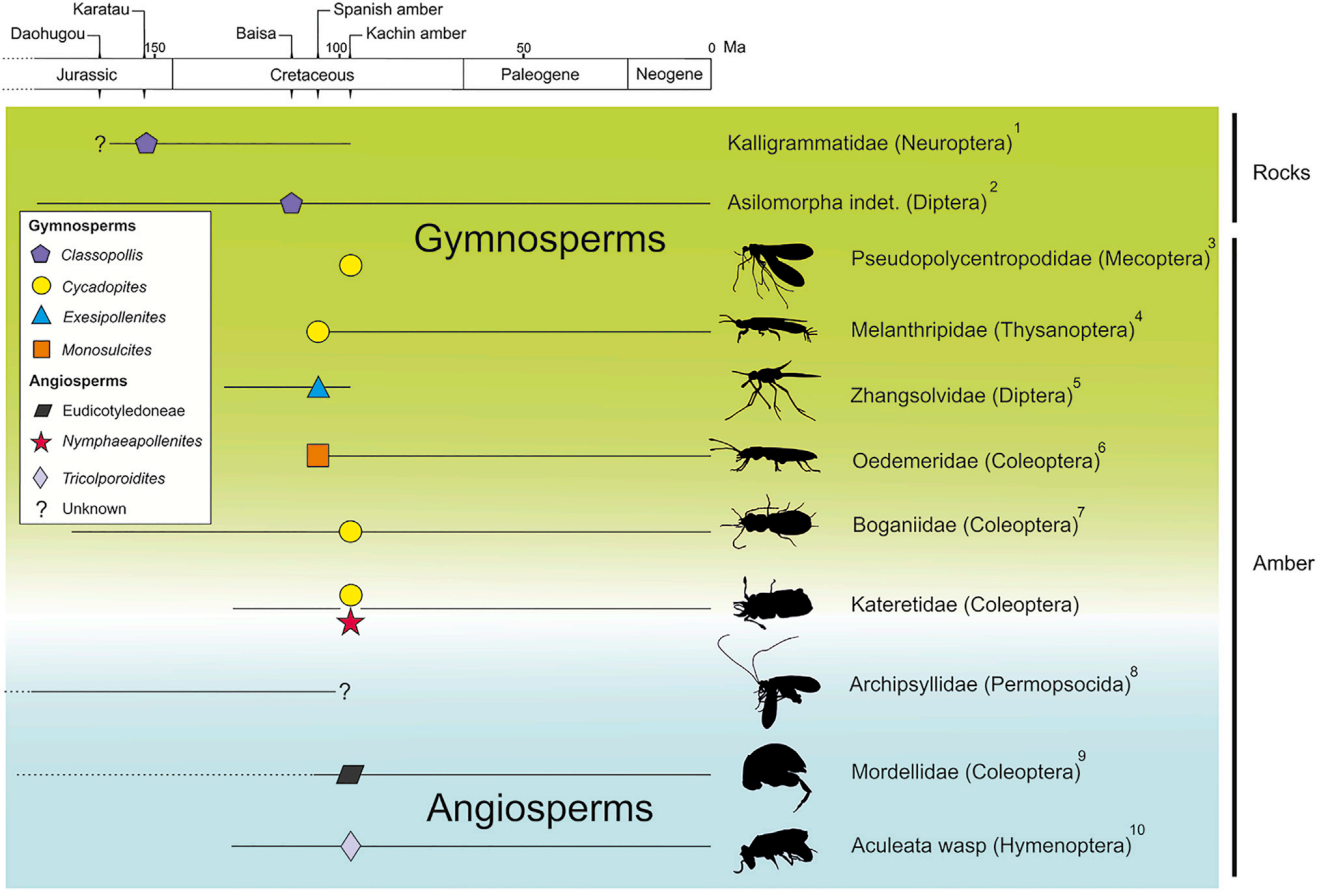
The Geochronological Ranges of Mesozoic Insect Taxa Exhibiting Direct Evidence of Pollination Geometric figures indicate the taxonomic affiliations of the pollen types found in each example. Superscript numbers: 1 (Labandeira et al., 2016); 2 (Labandeira, 2005); 3 (Lin et al., 2019); 4 (Peñalver et al., 2012); 5 (Peñalver et al., 2015); 6 (Peris et al., 2017); 7 (Cai et al., 2018); 8 (Huang et al., 2016); 9 (Bao et al., 2019); 10 (Grimaldi et al., 2019). The different colors in the background illustrate the gymnosperm/angiosperm origin of the pollen grains found. Kateretidae is located in the transition between both group of plants as some samples were found associated with gymnosperms and also one sample with angiosperms.

Direct evidence of angiosperm pollination by insects was only known from the Cenozoic (Labandeira, 1997, Wappler et al., 2015) until recent finds from Myanmar amber (Huang et al., 2016, Grimaldi et al., 2019, Bao et al., 2019; this work). Three reports of Cretaceous insects with pollen (Caldas et al., 1989, Poinar, 2010, Sendi et al., 2020) are difficult to evaluate (Grimaldi et al., 2019). A xyelid sawfly from the Crato Formation of Brazil was identified with pollen of *Afropollis* (Caldas et al., 1989). Regrettably, the record comes from a meeting abstract without images or other documentation and the efforts from David Grimaldi (American Museum of Natural Museum, NY) to find the specimen in Brazil failed (Grimaldi et al., 2019). The other records are a fly from Myanmar amber (Poinar, 2010) and several cockroaches from Lebanon and Myanmar ambers (Sendi et al., 2020). These works reported putative pollen, lacking detail necessary to determine whether these grains are not debris particles or bubbles, according to the low resolution and the different sizes hinted at the images. In compression deposits from Daohugou, China (165 mya), and Karatau, Kazakhstan (155 mya), two long-proboscid neuropterans of (fossil family)Kalligrammatidae with *Classopollis* and unknown pollen grains, respectively, were found in contact with particular regions of the head (Labandeira et al., 2016). One undetermined fly of Asilomorpha received clusters of *Classopollis* pollen smeared onto its mouthparts, from Baissa, Russia (130–105 mya) (Labandeira, 2005). In 105-million-year-old Spanish amber there are four female thrips specimens (Thysanoptera: Melanthripidae) covered by *Cycadopites*-type pollen grains, attributed to a ginkgoalean or possibly cycadalean host (Peñalver et al., 2012). A long-proboscid fly of ^†^Zhangsolvidae carried an *Exesipollenites* pollen clump, most likely belonging to a bennettitalean (Peñalver et al., 2015). An Oedemeridae beetle was associated with *Monosulcites* pollen grains (Peris et al., 2017). In addition, in 99-million-year-old Myanmar amber, a beetle assigned to Boganiidae was associated with *Cycadopites*-type pollen grains (Cai et al., 2018), and a long-proboscid scorpionfly of ^†^Pseudopolycentropodidae recently was described with associated *Cycadopites*-type pollen grains (Lin et al., 2019). Angiosperm pollen grains recovered from the gut and body surface of a permopsocid from the same amber deposit was interpreted as a strict case of pollinivory (Huang et al., 2016), although the authors did not indicate whether there was the presence of pollination. Very recently, an aculeata wasp was found together with numerous eudicot pollen grains from the genus *Tricolporoidites* (Grimaldi et al., 2019) and a mordellid beetle with eudicot tricolpate grains (Bao et al., 2019), both also from Myanmar. Although authors in the first case make an extensive and multidisciplinary vision of what the find represents (Grimaldi et al., 2019), description of the mordellid specimen can be improved and some characters may be corrected after the examination the holotype by the first author. It is denoted that all the unequivocal, recent documentation about insect-angiosperm pollen associations are provide from Myanmar amber (ca. 99 mya), together also with many other examples of associations with gymnosperms (Figure 4). By contrast, more ancient records (including those in Spanish amber) are always associated with gymnosperms up to now (Figure 4).

### History and Biology of the Kateretidae

Kateretidae (short-winged flower beetles) are a family of beetles (Coleoptera), members of the suborder Polyphaga, which range from small to medium in size and at present consist of 14 genera and approximately 95 species occurring in subtropical to temperate zones of the northern and southern hemispheres (Jelínek and Cline, 2010). Larvae occur in flowers of angiosperms, where the documented host plants commonly are Papaveraceae (poppies), and also members of Scrophulariaceae (figworts), Urticaceae (nettles), and Caprifoliaceae (honeysuckles, teasels, and valerians) among dicots and Asparagaceae (yuccas), Cyperaceae (sedges), and Juncaceae (rushes) among monocots (Jelínek and Cline, 2010). The larval host plants of six genera remain unknown. Typically, larvae feed on anther sacs and consume ovaries and seeds of the same flower. Adults occur on their larval host plants for mating and oviposition, but have a more generalized range of hosts for feeding, especially Rosaceae (roses, plums, and relatives), and also Asteraceae (sunflowers) and Apiaceae (umbellifers) (Kirk-Spriggs, 1996). The oldest fossil kateretid was described in the Early Cretaceous Lebanese amber (Kirejtshuk and Azar, 2008), and the family is also known from Myanmar amber (Poinar and Brown, 2018) and other younger deposits. Specimens from the four samples reported here display sufficient autapomorphic characters to be assigned minimally to four separate genera (Peris and Jelínek, 2019, Peris and Jelínek, 2020). Based on occurrence data, it appears that Kateretidae were widespread and abundant amid the early Cenomanian forest of Myanmar, where they were visiting diverse seed plants (Peris and Jelínek, 2019).

### History and Biology of Nympheaceae

The Nymphaeaceae (water lily) together with the Cabombaceae (water shields) and the Hydatellaceae, constitutes the Nymphaeales, a clade of aquatic plants that is a sister group or adjacent paraphyletic group to extant Amborellaceae (Friis et al., 2001, Soltis et al., 2018), the phylogenetically basalmost angiosperm lineage (Friis et al., 2001, APG, 2009). However, at present its monophyly represents a critical question (Gruenstaeudl, 2019). Compared with other angiosperm plant lineages of the Cretaceous, the water lilies have a modest fossil record. The oldest definitive fossil flower of Nymphaeaceae is described from a well-preserved charcoalified flower from the late Aptian to early Albian Vale de Agua locality in Portugal (Mohr et al., 2008, Friis et al., 2009). Notably, the apomorphic features of this earliest fossil occurrence is referred to the crown group of Nymphaeaceae, indicating that other lineages within the Nymphaeales, such as Cabombaceae and Hydatellaceae, had diverged earlier in the Cretaceous from an *Amborella*-like lineage, probably during the Barremian to early Aptian interval (Friis et al., 2009). A younger fossil from the late Aptian to early Albian Crato Formation of Brazil (Friis et al., 2000) has been affiliated with the Nymphaeaceae, although it likely is more closely related to the sister group Cabombaceae (Friis et al., 2009). Likewise, there are other Cretaceous occurrences of fossils assigned to Nympheaceae in compression deposits, such as the aforementioned locality in Portugal, the late Barremian to early Aptian of Virginia in the United States (Friis et al., 2000, Friis et al., 2011), Albian of Jordan (Taylor et al., 2008), Turonian of the southern Negev Desert in Israel (Dobruskina, 1997, Krassilov et al., 2005), and the early Santonian of northeastern Honshu in Japan (Takahashi et al., 2007). However, these occurrences are best assigned to Cabombaceae or undetermined Nymphaeales (Friis et al., 2011). Although Nympheaceae is represented by few occurrences in the Cretaceous, the family is more abundant during the Cenozoic, in which a distinctive seed record supports the presence of modern genera of Nymphaeaceae (Reid and Chandler, 1938, Dorofeev, 1974, Collinson, 1980, Mai, 1995).

Nympheaceae are currently herbaceous insect-pollinated angiosperms defined by a distinctive morphology. They possess rhizomatous roots; stems that bear vascular bundles, prominent air-filled canals, often lactifers, and simple, mucilage-producing hairs; and leaves that are typically whorled on the stem and have long petioles that are submerged, floating, or emergent with palmate or pinnate venation (Judd et al., 1999). The flowers are large, conspicuous, and radially symmetrical, consisting of four to twelve separate tepals that frequently are petal-like, scented, and produce a nectar-like secretion and have eight to numerous inner staminodes (sterile stamens) that are bladelike, conspicuous, and resemble true petals (Willemstein, 1987). The innermost fertile stamens often are attached to these staminodes and bear two, distinct, elongate, valvate filaments arranged longitudinally at their tips (Judd et al., 1999, Soltis et al., 2018). The pollen is globose-oblate, globose-spherical, or boat-shaped with anasulcate or zonasulculate apertures (Sampson, 2000). Peripherally lobed stigmas are elongate to radiate and occur as a circular disk centrally positioned in the flower. The stigma typically produces a carbohydrate-rich secretion, although nectaries sometimes are present nearby on the staminodes (Judd et al., 1999, Bernardello, 2007). For Nymphaeaceae, one to numerous anatropous to orthotropous ovules are positioned variously on the parietal placenta that forms three to many distinct or connately fused carpels. The fruit is an aggregate of nutlets or indehiscent pods, or alternatively a berry or an indehiscent capsule. The seeds are open by means of a cap-like structure and lack endosperm, although an abundant perisperm is present.

Extant water lilies occur worldwide in temperate to tropical climates in standing freshwater ecosystems (Schneider and Williamson, 1993, Borsch et al., 2008). Such an environment is consistent with recent findings of marine and freshwater fossils in Myanmar amber, which suggests that the local resinous forest was present adjacent to a coastal embayment or a deltaic environment (Schmidt et al., 2018, Xing et al., 2018, Yu et al., 2019). The hydrophyte niche occupied by Nymphaeaceae extended deep into the Early Cretaceous (Friis et al., 2011) and probably included attractants such as petaloid floral structures with embedded nectar, emission of fruity odors, and receptacular thermogenesis that sequester pollinating insects (Seymour and Matthews, 2006). Pollinators of Nymphaeaceae typically are insects of medium to large size, particularly beetles, flies, and bees, whose rewards principally are pollen and secondarily nectar or nectar-like secretions produced by most elements of the flower (Prance, 1980, Lippok et al., 2000, Hirthe and Porembski, 2003). For larger-flowered species, beetles especially are attracted to starch-laden food bodies that are appendages of the carpels and use as mating sites the heat generated in the receptacle portion of the flower along with the emission of strong, fruity odors (Judd et al., 1999, Prance, 1980, Hirthe and Porembski, 2003, Fava and Gomes, 2017). These attractants lure beetles while the protogynous flower is open, followed by closure that allows the trapped beetles to acquire or deposit pollen, respectively, in contact with the stamens and stigmas that often are partially consumed (Prance, 1980, Willmer, 2011). Documented insects that pollinate water lilies are especially large scarab beetles (Prance, 1980, Willemstein, 1987, Hirthe and Porembski, 2003; but see Meeuse and Schneider, 1979). In other, smaller-flowered species, smaller, pollen-bearing insects of beetles, flies, and bees obtain pollen from flowers that are a few days old and are attracted to abundant, dilute, and carbohydrate-rich stigmatic exudations that pool on the discoidal stigma (Schmucker, 1932, Meeuse and Schneider, 1979, Schneider and Chaney, 1981, Schneider, 1982, Judd et al., 1999). The insects drown in this pool, liberating the pollen on their bodies and resulting in ovular fertilization; subsequently, the flower enters a pollen phase in which the stamens form a closed cone that denies access to the stigma (Faegri and van der Pijl, 1980, Schneider and Chaney, 1981, Judd et al., 1999).

### Evolution of the Pollination Mutualism between Insects and Plants

Of the four major lineages of current gymnosperms, wind pollination is the exclusive pollination mode in extant conifers and *Ginkgo* (Nepi et al., 2017), whereas insects overwhelmingly are pollinators of cycads and gnetaleans (Labandeira, 2010, Ickert-Bond and Renner, 2016). For the latter, pollinator assemblages vary in their specialization and range from near-obligate mutualisms in some cycads to eclectic, generalized associations in almost all gnetaleans (Labandeira et al., 2007, Nepi et al., 2017). Most seed-plant species are visited by taxonomically diverse groups of pollinators, and pollinator species overwhelmingly visit several or more plant species (Waser et al., 1996). It has been established that insects were consuming pollen that considerably antedated the evolution of angiosperm traits for attracting and rewarding pollinators (Labandeira et al., 2007). Entomophily was then a plesiotypic condition for angiosperms (Hu et al., 2008, Thien et al., 2009). Crepet and Friis (1987) supported the hypothesis that insect pollination evolved from Carboniferous seed ferns and subsequently was extended to Bennettitales during the Jurassic and then to magnoliacean (magnolias) and annonacean (custard apples) angiosperms in the Early Cretaceous. Gymnosperm pollen or secretions like the pollination drops fed insects as awards initially (Grimaldi, 1999, Labandeira, 2010, Nepi et al., 2017). Long-proboscid mouthpart structures known from the Mesozoic required deep-throated receiving structures in ovulate organs of gymnosperms; the bowl-shaped flower structure of early angiosperms likely was pollinated by unspecialized, small, mandibulate insects such as beetles (Dilcher, 2000, Endress and Igersheim, 2000, Wang et al., 2013, Labandeira et al., 2016, Lin et al., 2019). Notably, the distinctive, tubular, floral modifications of angiosperms that would accommodate long-proboscid insect pollinators originated during the Paleogene (Labandeira, 2010, Friis et al., 2011, Lin et al., 2019). The extinction of several long-proboscid lineages during the Cretaceous is consistent with this pattern, as Cretaceous angiosperms lacked relevant floral structures that would accommodate long-proboscid insects (Ren et al., 2009, Endress, 2010, Labandeira, 2010, Peñalver et al., 2015, Lu et al., 2016, Liu et al., 2018, Lin et al., 2019). In distinct contrast to the extinction of many long-proboscid insect pollinators, other mandibulate pollinators, including beetles, made the transition from gymnosperm to angiosperm hosts with greater facility. After these transitions, the Nympheaceae (water lilies) would have been among the most likely candidates for insect pollination being among the earliest angiosperms (but see above). Pollen grains of early angiosperms initially were dry, with an absence of sticky substances such as pollenkitt and viscin threads to cause pollen clumping (Hu et al., 2012). The evolution of pollen clumping resulted in more efficient pollination and changes in fertilization patterns such as evolution of syncarpous ovaries (Friis et al., 2006, Thien et al., 2009, Taylor and Hu, 2010). By Cenomanian time, commencing at 100 mya, features of flowers such as nectaries and pollen specialized for insect transport were present (Crepet and Friis, 1987, Friis et al., 2006, Friis et al., 2011, Hu et al., 2008, Hu et al., 2012, Taylor and Hu, 2010).

### Conclusions

**Figure 5:**
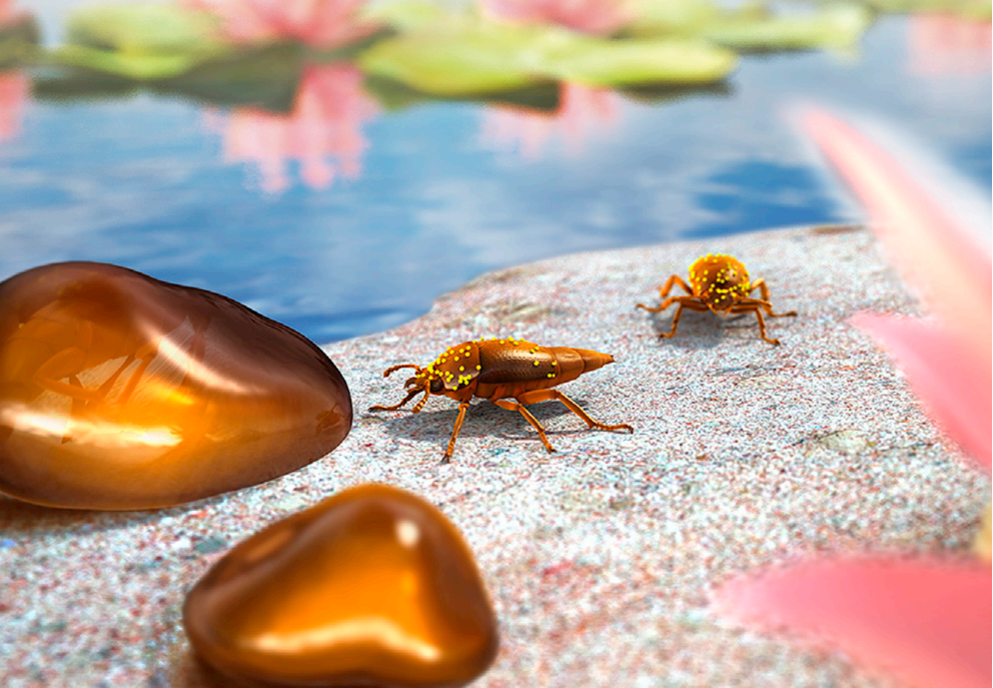
Reconstruction of Kateretid Beetles Two specimens of *Polliniretes penalveri* from the sample NIGP171365 with nymphaeacean pollen grains of *Praenymphaeapollenites cenomaniensis* gen. and sp. nov. attached to their body surfaces. Two viscous blobs of resin are nearby. Art by J.A. Peñas.

Early angiosperms evolved in an environment where small insects such as moths, thrips, beetles, flies, and wasps were feeding on a variety of seed-plant lineages as the sole hosts, including ginkgoaleans, bennettitaleans, and cycads (Gottsberger, 1988, Labandeira et al., 2007, Peris et al., 2017). The relationship between beetles and water lilies already occurred during the Early Cretaceous (Figure 5), in which there were a pre-existing guild of beetles that pollinated gymnosperms (Gottsberger, 1988, Labandeira et al., 2007, Ervik and Knudsen, 2003, Peris et al., 2017, Cai et al., 2018, this work). The transition of beetle pollinators from an older gymnosperm to a more recent angiosperm host was described for Oedemeridae (Peris et al., 2017) and now is observed with direct associational evidence for Kateretidae. The origin of angiosperm pollination among polylectic beetle pollinators is essential for understanding not only early pollination strategies but also the rapid success of flowering plants during the mid-Cretaceous.

### Limitations of the Study

The study of pollen grains requires observation under high-magnification lenses; however, most pollen grains are badly preserved and important characters of the grains are hardly found. The observation of different grains most exposed at the amber surface was required to completely describe the pollen characters. Furthermore, several grains are in contact with the body surface of the specimens, although in areas with limited access to be illustrated.

## Methods

All methods can be found in the accompanying Transparent Methods supplemental file.

## Supplemental Information

Document S1. Transparent Methods, Figures S1–S3, and Table S1
