# Supplementary material for: Generalist Pollen-Feeding Beetles during the Mid-Cretaceous
Source: iScience. 2020 Mar 18;23(3):100913. doi: 10.1016/j.isci.2020.100913 (PMC7113562; doi:10.1016/j.isci.2020.100913)
Supplement: Document S1. Transparent Methods, Figures S1–S3, and Table S1 [file mmc1.pdf]

iScience, Volume ■ ■

Supplemental Information

Generalist Pollen-Feeding Beetles

during the Mid-Cretaceous

David Peris, Conrad C. Labandeira, Eduardo Barrón, Xavier Delclòs, Jes Rust, and Bo Wang

Supplemental data items

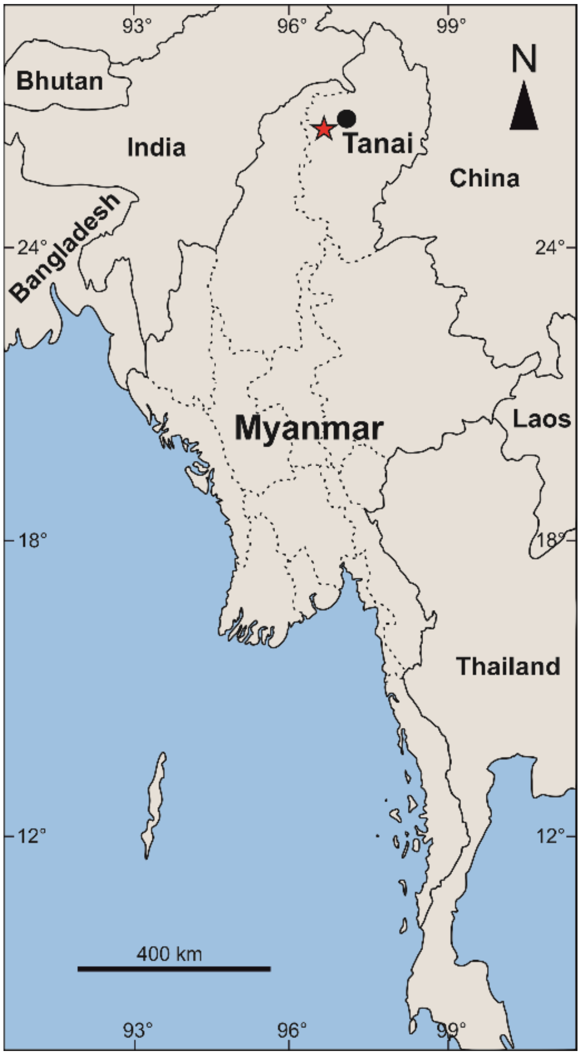

Figure S1. Location of the amber mine near Tanai (= Danai) in Kachin State, northern Myanmar; early Cenomanian, ~99 million years in age. Related to Figures 1 and 2.

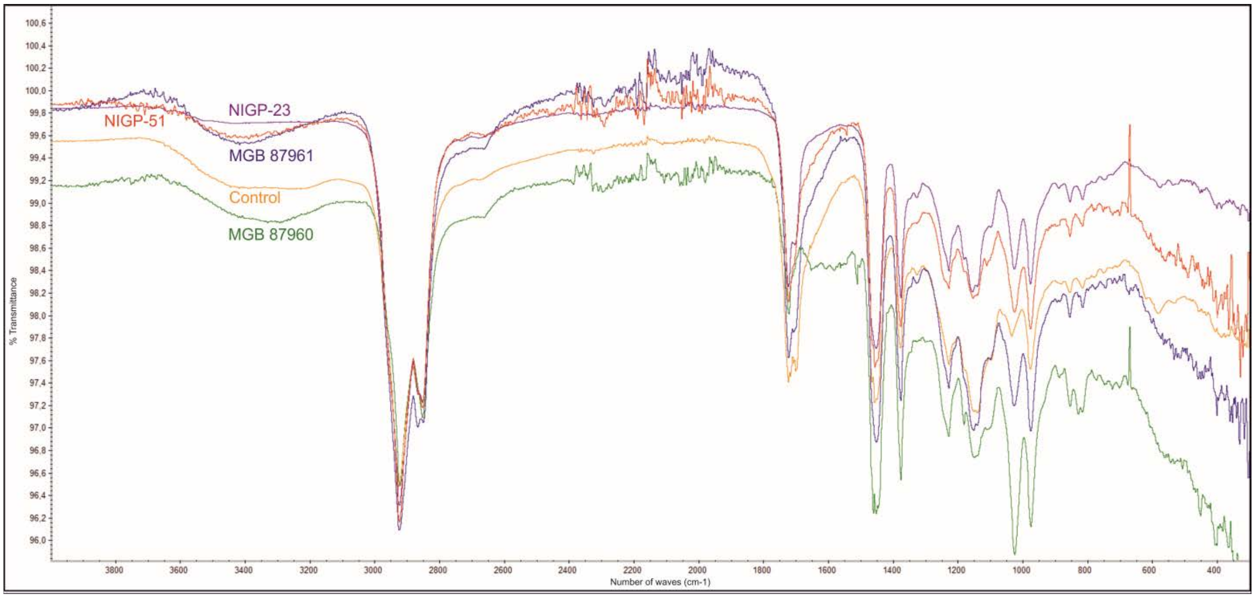

Figure S2. Fourier transform infrared spectroscopy (FTIR) analyses of the four pieces of amber studied herein and a control sample. All the analyzed samples show a similar spectra, although with different signal intensity. Related to Figure 1.

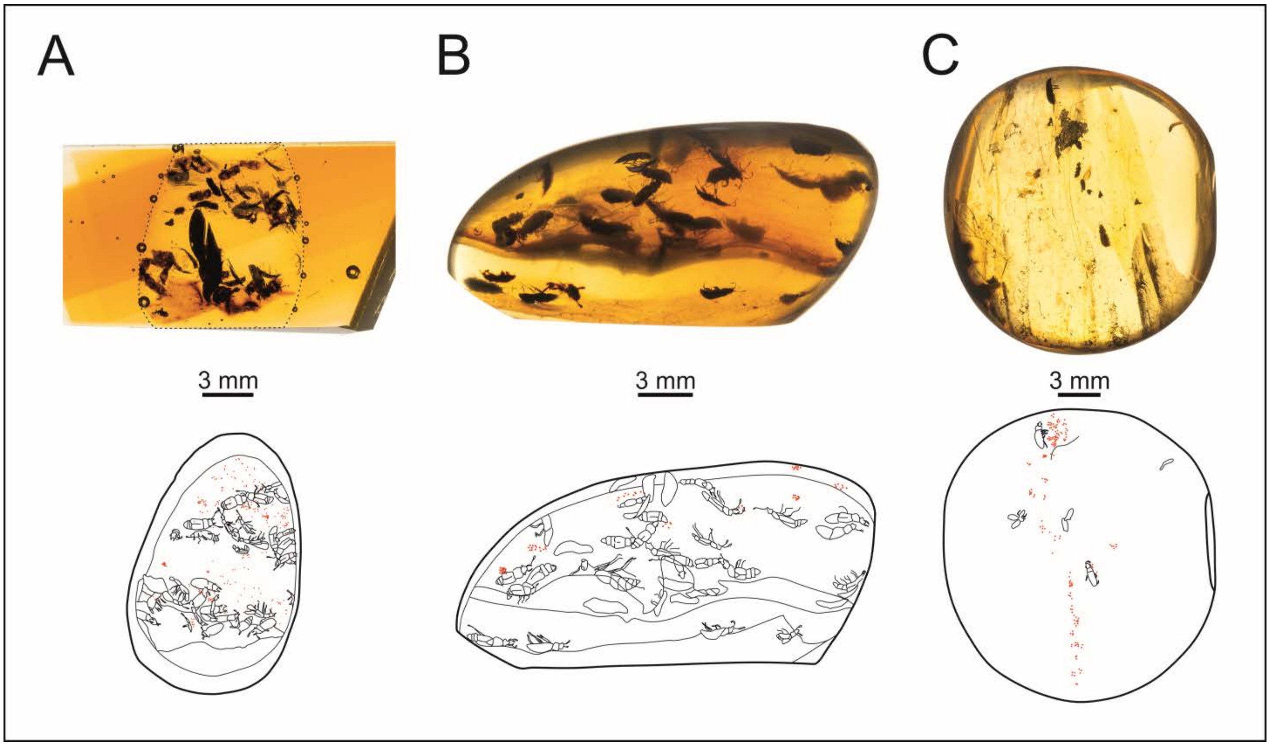

Figure S3. Light microscope photograph and their facsimile camera lucida drawings of the amber samples described in this work, except for NIGP171365 (in Fig. 1). Each pollen grain is represented by a red dot placed in its observed position within the amber sample. A, Sample MGB 87960. B, Sample MGB 87961. C, Sample NIGP171364. Related to Figures 1 and 2.

Table S1. Length and width measurements of pollen grains. Related to Figure 2.

**Gymnosperm**						**Angiosperm**
**MGB 87960**		**MGB 87961**		**NIGP171364**		**NIGP171365**
Length (μm)	Width (μm)	Length (μm)	Width (μm)	Length (μm)	Width (μm)	Diameter (μm)
20.71	18.57	17.85	10.71	24.28	15	50
21.43	14.28	20	12.85	23.57	10.71	60. 71
21.44	17.14	15.71	9.28	21.42	11.43	53.57
20	17.85	12.85	7.14	25	14.28	67.85
22.85	16.43	17.14	11.43	25	14.28	62.85
20.71	22.85	21.42	7.14	24.28	14.28	64.28
22.14	15.71	19.28	12.85	27.85	16.43	71.42
21.43	17.85	21.42	8.57	24.28	14.28	62.14
20.71	16.43	17.85	12.85	31.43	14.28	60
18.57	18.57	17.85	8.57	30	15.71	60.71
21.43	18.57	20	10.71	26.43	15	60
20	22.85	19.28	12.85	28.57	16.43	55
21.43	22.85			28.57	10.71	52.14
18.57	15			32.14	20	67.85
21.43	21.44			27.85	15.71	42.85
				26.43	17.85	69.28
				25	15.71	64.28
				24.28	12.85	48.57
				25	12.85	64.28
				25	17.14	52.14
				31.43	12.85	46.42
				25.71	12.85	64.28
				22.85	17.85	51.14
				24.28	17.85	78.57
						53.57
						60

						85.71
						64.28
						55
						71.42
Average	Average	Average	Average	Average	Average	Average
20.89 (18.57-22.85)	18.42 (14.28-22.85)	18,41 (15.71-21.42)	10.41 (7.14-12.85)	26.27 (21.42-32.14)	14.84 (10.71-17.85)	60.67<br><br>(42.85–85.71)

Transparent Methods

The examined specimens were found in four separate pieces of Myanmar amber from the Tanai (=Danai) locality in Kachin State (Fig. S1), of Late Cretaceous (early Cenomanian) age. All pieces were cut and polished, and sample MGB 87960, originally oval, initially was embedded in transparent Araldite 2020 resin, following the procedure outlined in Nascimbene and Silverstein (2000). Samples MGB 87960 and MGB 87961 are housed at the Laboratori de Natura of the Museu de Ciències Naturals de Barcelona (Barcelona, Spain). Samples NIGP171364 and NIGP171365 are housed at the Nanjing Institute of Geology and Palaeontology, Chinese Academy of Sciences, Nanjing, China. The beetle and pollen grains were examined with a Leica MS5 and a Leica MZ95 stereomicroscope, as well as a Motic BA310, an Olympus BX51 and Olympus CX41 compound microscopes. Drawings were made under incident light with the aid of a camera lucida attached to a Leica MZ95 stereomicroscope and later were inked and scanned. General photomicrographs of the samples were created with a Nikon D3X and an objective AF-S Micro-NIKKOR 60 mm 1:2,8 G ED. Microphotographs of the pollen grains were taken by a Moticam 2500 camera on a Motic BA310 microscope and a ColorView IIIu camera attached to an Olympus BX51 compound microscope. Photomicrographs of the pollen in contact with the beetles were taken by a Euromex sCMEX camera on an Olympus CX41 compound microscope. A Fourier transform infrared spectroscopy (FTIR) analyses of the four pieces of amber studied was conducted in the Scientific Facilities of the University of Barcelona in order to analyze the provenance of the material (Fig. S2). The FTIR results were compared with a control piece found in Tanai (type locality in Myanmar) 20 years ago. We used a diamond PerkinElmer FT-IR Spectrometer Frontier; software version 10.4.2 (2014). All the figures were edited using CorelDraw-X8 software.

Supplemental References

Nascimbene, P., and Silverstein, H. (2000). The preparation of fragile Cretaceous ambers for conservation and study of organismal inclusions. In Studies on Fossils in Amber, with Particular Reference to the Cretaceous of New Jersey, D. Grimaldi, ed. (Backhuys), pp. 93–102.
